# Paying for Health Gains Using Patient Reported Outcome Measures

**DOI:** 10.1002/hec.70086

**Published:** 2026-02-23

**Authors:** Luigi Siciliani, James Gaughan, Nils Gutacker, Hugh Gravelle, Martin Chalkley

**Affiliations:** ^1^ Department of Economics and Related Studies, and Centre for Health Economics University of York York UK; ^2^ Centre for Health Economics University of York York UK

**Keywords:** health, hospitals, pay for performance, quality

## Abstract

Payments to healthcare providers are often based on the number of patients with a particular diagnosis or treatment with well known limitations. Payment based on health outcomes, a form of pay‐for‐performance, has long been advocated as a possible solution. We use a contract theory approach and illustrate how it can inform practical implementation of pay‐for‐performance schemes that reward health outcomes. The pricing rule suggests that the bonus should be set to reflect the difference between the provider's marginal cost of a health improvement before the policy intervention and the provider's marginal cost evaluated at the target health set by the purchaser. We provide estimates of the optimal bonus for hip and knee replacement under a range of assumptions about provider cost functions and the value of health improvements.

## 1 Introduction

Reimbursement to healthcare providers are often based on the number of patients with a particular diagnosis or treatment. Activity‐based financing based on Diagnosis Related Groups (DRGs) remains dominant across OECD countries. There are however a number of concerns with these payment mechanisms including under‐treatment, if providers skimp on quality, or over‐treatment if rewarding activity induces providers to engage in costly activity that does not improve health. The fundamental issue is that payment based on activity alone does not take into account the value of treatments being provided. One intuitive response is to make prices reflect the additional value of the health outcomes produced. This would require measuring the outcome of treatment in terms of health gain and paying a bonus that rewards health improvements. Payment based on health outcomes has long been discussed and sometimes advocated as a natural development of activity‐based financing (Dunbar‐Rees 2018).

The appeal of outcome‐based payment systems is that they reward what ultimately matters, health gains. However, health outcomes are difficult to measure. Some commonly available indicators, such as mortality and readmission, only apply to treatments where there is a sufficient incidence of failure and high volume. Moreover, both mortality and readmission rates measure health on one tail of the outcome distribution. More recently, broader measures of health gains have been collected in the form of patient‐reported outcome measures (PROMs) for common procedures such as hip and knee replacement (NHS England 2013; Gutacker et al. 2013). These measures consider health gains across the whole distribution.

Even with accurate health measures, there is lack of consensus on how the incentive bonus should be set. The evidence suggests that providers, hospitals in particular, tend to have limited response to pay‐for‐performance schemes (Cashin et al. 2014; Milstein and Schreyoegg 2016; Mendelson et al. 2017).^1^ A possible explanation is that the size of the bonus was relatively small (about 5% of hospital revenues with some variation across schemes and countries (Cashin et al. 2014)), and so insufficient to induce changes in provider behavior.^2^

This study adopts a contract theory approach to examine the appropriate size of the bonus to inform practical implementation of pay‐for‐performance (P4P) schemes that reward health outcomes. First, we provide a simple but general model for the design of an incentive scheme that rewards health gains, based on key parameters related to patient health benefits and provider costs (Section 2). Second, the model is calibrated with health outcome and cost data from two elective procedures, hip and knee replacement, to illustrate the applicability of the framework and show the sensitivity of the bonus to different assumptions on benefits and costs (Section 3). The study bridges the gap between economic theory and policy, and informs the design of P4P schemes where health is observable and measured accurately. The model takes a representative provider approach. We show that the findings are robust to extending the analysis to heterogeneous providers (Section 4).

In line with the literature (Ellis and McGuire 1986; Chalkley and Malcomson 1998), we assume that providers are altruistically or intrinsically motivated and choose a level of quality that trades off the benefits from higher quality against its costs. We then model the purchaser decision regarding the bonus. We first adopt a positive economics perspective where the purchaser specifies a bonus to achieve a health objective or target based on the observed empirical distribution across providers (e.g., increasing the average health gain by one standard deviation or to the level of health in the top quartile or quintile of the health distribution).

We then adopt a cost‐effectiveness analysis approach and provide a framework to explore whether the introduction of the bonus is cost‐effective. We compare the value of the health benefits generated by the scheme against the additional provider costs, which in our model coincide with the additional reimbursement made by the funder of health services (i.e., the National Health Service). Finally, we use a welfare function approach and derive the optimal pricing rule that induces a level of health which maximizes the difference between the value of patient benefits and provider costs. We also relate this approach to cost‐effectiveness analysis.

Throughout the analysis, we assume that the bonus is in addition to the basic activity‐based price (Healthcare Resource Groups (HRG) tariff, the English equivalent to DRG tariff), which can be adjusted to ensure that provider revenues cover treatment costs. This implies that the amount reimbursed by the purchaser to the provider is always equal to the provider's costs. This approach is broadly in line with several health systems that use DRGs as basis for reimbursement. The DRG tariff is generally based on past national average hospital costs for a specific diagnosis/treatment (with a one‐ or 2‐year lag): a form of yardstick competition (Shleifer 1985). In England, the introduction of P4P schemes for hospitals (known as Best Practice Tariffs) have combined the introduction of the bonus with a reduction of the basic HRG tariff. This was the case for Best Practice Tariffs that incentivized quality for stroke (Kristensen et al. 2016) and hip fracture (Grašič et al. 2025).

Given our applied focus, we express the bonus as a function of the post‐operative health *before* the introduction of the bonus, and the target post‐operative health *after* the introduction of the bonus, as this information is available or chosen by the purchaser. In our positive analysis, we show that the optimal pricing rule for the purchaser is to choose a bonus that reflects the difference between the marginal cost of post‐operative health before the policy intervention and the marginal cost of post‐operative health at the target level of health set by the purchaser (i.e.., post policy).

We show that that the incentive scheme is cost‐effective if the cost‐effectiveness ratio is below a given threshold, where additional health benefits are compared to additional provider costs, which in our model coincide with the additional reimbursement to the provider. Under a welfare‐function approach, we also show that the optimal price per unit of health is equal to the marginal benefit from health, weighted by the opportunity cost of public funds, net of provider marginal cost of health before the introduction of the bonus payment.

Using data from hip and knee replacement in England we calibrate the model for the average provider with respect to two key parameters, provider costs and post‐operative health. We compute the bonus payments that the purchaser would have to make to achieve target levels of post‐operative health equivalent to improvements of one or two standard deviations of the health distribution observed across providers. To infer the shape of the cost function for the average provider, we make assumptions related to fixed and variable costs. In our calibration for hip replacement, we find that the price for one unit of health improvement as measured by the Oxford Hip Score (OHS) to achieve an improvement of 1.13 points on the OHS scale (equivalent to one standard deviation observed in the empirical distribution) ranges between £45 and £226 under different assumptions related to the cost function. For knee replacement, the price for one unit of health improvement as measured by the Oxford Knee Score (OKS) to achieve an improvement of 1.06 points on the OKS scale (equivalent to one standard deviation observed in the empirical distribution) ranges between £72 and £254 under different assumptions related to the cost function. The price doubles for a health target of two, rather than one, standard deviation improvement.

When we evaluate the health benefit in quality‐adjusted life years (QALYs), for which there are official estimates of the purchaser willingness‐to‐pay in the English NHS, we generally find that the introduction of the bonus is cost effective, even at high levels of health targets. In turn, this implies that under a welfare function approach, the optimal health target is high and so is the price per unit of health improvement.

Our analysis makes explicit which cost and benefit parameters are required for practical implementation of paying for health schemes. Even for a positive analysis where the purchaser sets an ad hoc health target, the approach requires assumptions about the marginal cost of health improvement both at the status quo and at the target level of health. One approach is to take an accounting perspective and gather data and estimate the cost of plausible hospital interventions to increase post‐operative health (e.g., through an estimate of the time that health workers would have to devote to implement such interventions). Another approach is to conduct empirical analyses to estimate the marginal cost of increasing health for specific procedures.

### 1.1 Related Literature

There is a relatively small literature which calibrates theoretical models of incentive schemes for healthcare providers. The closest study is Kristensen et al. (2016) that presents a stylized model for hospital price setting for (three) process measures of quality. The model is calibrated using features of the Best Practice Tariffs scheme for emergency stroke care in the English NHS that rewarded hospitals for ensuring treatment in an acute stroke unit, rapid brain imaging, and provision of thrombolytic medication to some patients. Our study differs in several ways. First, we investigate pricing schemes that stimulate quality by targeting health outcomes, rather than process measures of quality. Second, we derive the pricing rule as a function of pre‐policy intervention parameters. Third, we include a positive analysis and a cost‐effectiveness approach, in addition to a welfare analysis. Finally, our calibration applies to elective rather than emergency care.

Lisi et al. (2020) provide a theoretical analysis of P4P to encourage reductions in mortality and readmission rates when patients differ in severity, hospitals compete on quality, and risk‐adjustment is incomplete. They show that P4P can weaken or strengthen hospitals' incentive to provide quality. Since patients with higher severity are more likely to exercise choice, this introduces a patient composition effect which alters quality incentives and is stronger in more competitive environments. They calibrate the model based on the Premier Hospital Quality Incentive Demonstration introduced by the Centers for Medicare & Medicaid Services in 2012 and the Hospital Readmission Reduction Program. Unlike our paper, no optimal pricing rule is derived. Sa et al. (2019) model hospital competition in the presence of excess demand and waiting times. They calibrate the model using waiting times and volume for cataract surgery in the English NHS to compare different solution concepts, but again no optimal pricing is derived.

Chalkley and Malcomson (2003) investigate the scope for refining DRG hospital payments by allowing for cost sharing. They show that if providers differ in costs and there is asymmetric information, then total payment can be reduced by cost sharing. Calibration with Medicare data suggests that cost sharing can generate cost savings that range from 7% for treatments with low‐cost variation to 60% for those with high‐cost variation. By contrast, we assume that costs cannot be contracted on.

There is a larger theoretical literature investigating provider incentives in relation to quality and cost containment, which derives qualitative insights on provider behavior and the features of optimal payment systems under a range of assumptions (see seminal studies by Ellis and McGuire 1986; Chalkley and Malcomson 1998; Siciliani 2018, for a review). A smaller literature focuses specifically on P4P, in relation to multitasking (tunnel vision) and gaming or misreporting (see Eijkenaar 2013, for an overview of key design issues). Eggleston (2005) shows that targeting some quality dimensions comes at the cost of reducing untargeted quality dimensions when qualities are substitutes, though cost sharing can mitigate this. Kaarboe and Siciliani (2011) show that this implies that the price is reduced as a result. Sherry (2016) finds that when P4P programmes reward multiple healthcare services in a setting characterized by joint production, the impact on both rewarded and unrewarded services is ambiguous. Kuhn and Siciliani (2009),  (2013) show that gaming also generally leads to lower‐powered incentive schemes. Mak (2018) studies a managed healthcare market with two differentiated hospitals. It analyses the interaction of P4P schemes on contractible quality with other features of the market, such as copayment and consumers' misperception of quality. These studies do not provide calibrations, which is a key focus of our paper.

## 2 Model

We adopt a contract theory approach (Ellis and McGuire 1986; Chalkley and Malcomson 1998) in which the principal (purchaser) can choose a payment mechanism that influences the choice of the agent (provider). Our focus is how the payment of a health‐related bonus affects the provider's decision concerning patient health gain from subsequent treatment.

In Section 2.1, we analyze the provider's choice of patient post‐operative health conditional on the payment rule. The provider therefore takes the reimbursement rule set by the purchaser as given. This consists of the basic DRG tariff and the bonus payment. We use a stylized setting of a representative provider that can expend costly effort to achieve better health outcomes. We implicitly assume that all patients are the same or, equivalently, that health measures are risk‐adjusted. This is important as the lack of risk adjustment could expose the provider to losses (or profits) that are due to external factors.

In Section 2.2, we consider the purchaser perspective under three different approaches. In designing the contract, the purchaser always anticipates the effect of the bonus payment on provider behavior. First, we consider how a purchaser can design a bonus to achieve a target level of health, which we label *positive analysis* (Section 2.2.1). Second, we investigate whether the introduction of a bonus is cost‐effective (Section 2.2.2). Third, we design a bonus which maximizes welfare (Section 2.2.3) defined as patient health benefits minus provider costs.

Formally, at time *t* = 1, the purchaser chooses the payment mechanism. At time *t* = 2, the hospital chooses the level of health provided to the patients. We solve by backward induction.

**Table:** 

Definition of variables	
h	Patient post‐operative health
$h_{0}$	Patient pre‐operative health
$\tilde{h}$	Threshold health level above which payment is received
$\underline{h}$	Level of health over which improving health is costly to the provider
$\hat{h}$	Purchaser health target objective
H(h)	Patient life‐time health gain
t	Prospective DRG/HRG fixed price
p	Bonus payment per unit of health improvement
C(h)	Provider cost function
k	Fixed treatment cost component
c	Parameter related to marginal cost of health improvement
α	Degree of provider motivation (altruism)
W	Willingness to pay for health improvement
δ	Opportunity cost of public funds
λ	Cost‐effectiveness threshold

### 2.1 The Provider Perspective

We define h as patient post‐operative health as determined by provider's effort to improve health and measured (at some point, e.g. six months) after the surgery.^3^ Pre‐operative health is $h_{0}$.

#### 2.1.1 Revenues

The provider is paid through a prospective Diagnosis Related Group (DRG) or Healthcare Resource Groups (HRG) tariff t and a health‐outcome related bonus p that is linear in health improvement.^4^ Hospital revenue R(h) is:

(1) $$R\left(h\right)=t+p\left(h-\tilde{h}\right),$$

where $\tilde{h}$ is a threshold health level decided by the purchaser and h is patient post‐operative health.

As an example, consider a patient in need of a hip replacement and that post‐operative health is measured with the Oxford Hip Score (OHS) on a 0–48 scale with higher values indicating better health. Suppose that the health threshold $\tilde{h}$ has an OHS of 30, that post‐operative health has a score of h=38, and that the price for one point of health improvement on the OHS scale is £100. Then hospital bonus payment from treating a patient, in addition to the HRG tariff, is $p\left(h-\tilde{h}\right)=100\left(38-30\right)=£800$. Equation (1) also encompasses the scenario where the provider has a penalty, rather than a bonus. This can be achieved by setting a threshold $\tilde{h}$ which is sufficiently high.^5^

The provider takes the HRG tariff t and the bonus p as given. This is plausible because the reimbursement rules are set by the purchaser at the beginning of the financial year. In Section 2.2, we discuss how the purchaser sets both the value of the tariff and the bonus payment.

#### 2.1.2 Costs

We assume that the provider has the following cost function for treating a patient:

(2) C(h)=k+K(h),

where k is a fixed treatment cost component, which is independent of health gain (e.g., the cost of an operating theater, the time of surgeons and nurses, anesthetist) and K(h) is the additional cost of improving the health of the patient, which includes both monetary costs (due to more expensive technology, medicines) and non‐monetary costs (e.g., time of nurses and doctors).^6^ We assume that the cost is increasing and convex in health. Higher levels of health increase costs, ∂K(h)/∂h>0, at an increasing rate, $\partial^{2}K\left(h\right)/\partial h^{2}>0$. While some health improvements are relatively easy to generate at low additional cost or even costless, it is increasingly costly for the provider to achieve additional health gains, which is captured by convexity of the cost function. For example, going beyond standard care would require additional rehabilitation, physiotherapy, follow‐up with the patient to ensure adherence with protocols and guidelines, etc.

To obtain a closed‐form solution, which we use for the calibration, we assume that the cost function is quadratic in h:

(3) $$C\left(h\right)=k+\frac{c}{2}{\left(h-\underline{h}\right)}^{2},$$

where c>0 is a parameter related to the marginal cost of a health improvement, and $\underline{h}$ is the level of health over which improving health is costly for the provider. We could set the latter at the pre‐operative health, $\underline{h}=h_{0}$. However, it is likely that providers with low level of costly efforts might still be able to achieve post‐operative health that is higher than the pre‐operative health. We therefore allow for the possibility that improving health up to $\underline{h}$ does not generate any additional costs on top of the fixed treatment costs k.^7^

#### 2.1.3 Motivation

We assume that the provider is motivated (due to altruism, intrinsic motivation toward quality or other reasons) and will improve the health of the patient even if not financially rewarded (as in Ellis and McGuire 1986; Chalkley and Malcomson 1998). We capture this motivation as B(h), which is increasing in health. We assume the following linear functional form:

(4) $$B\left(h\right)=\alpha \left(h-h_{0}\right),$$

where α is the degree of motivation. This parameter could also be interpreted more broadly as any source of motivation. In addition to altruism and intrinsic motivation, it encompasses other incentives to improve health that arise from monitoring and auditing mechanisms. For example, regulators can analyze quality indicators and conduct audits on a regular basis to ensure minimum standards. This parameter plays a critical role in the analysis, because higher levels of motivation imply higher levels of health, which reduces the scope to introduce financial incentives (as highlighted more comprehensively in Section 2.2). As illustrated below, if other regulatory mechanisms increase quality before the P4P bonus is introduced, the scope of P4P is reduced.

#### 2.1.4 Hospital Payoff Function

We assume that the payoff function of the provider V(h,p) from treating the representative patient includes both financial and non‐financial motives, and is additively separable in the motivation component B(h) and financial surplus π(h,p)=R(h,p)−C(h):

(5) $$V\left(h;p\right)=B\left(h\right)+\pi \left(h;p\right)=\alpha \left(h-h_{0}\right)+t+p\left(h-\tilde{h}\right)-k-\frac{c}{2}{\left(h-\underline{h}\right)}^{2}.$$

Maximizing with respect to the patient post‐operative health h, the provider chooses the level of health $h^{*}$ which satisfies the first‐order condition:

(6) $$\frac{\partial V\left(h;p\right)}{\partial h}=\frac{\partial B\left(h\right)}{\partial h}+\frac{\partial \pi \left(h;p\right)}{\partial h}=\alpha +p-c\left(h^{*}-\underline{h}\right)=0.$$

The provider balances the marginal monetary and non‐monetary benefits of increasing the patient's health against the marginal costs of health improvement. The second‐order condition is $\partial^{2}V\left(h;p\right)/\partial h^{2}=-c<0$. Changes in the basic DRG tariff t do not affect the level of health. This is because the tariff t is a lump‐sum payment that does not depend on the care provided. It is only the bonus payment per unit of health p that affects the patient's level of health. Higher levels of motivation α increase post‐operative health, while a higher marginal cost c reduces health.

**FIGURE 1:**
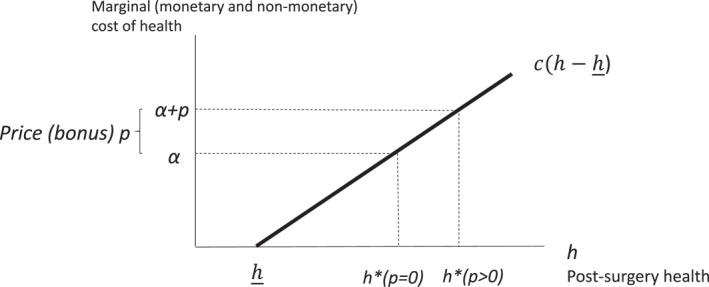
Provider choice of post‐surgery health.

The hospital's choice of patient post‐operative health is:

(7) $$h^{*}\left(p\right)=\underline{h}+\frac{p+\alpha }{c},$$

which is increasing in the bonus payment p.^8^ Figure 1 illustrates the solution, and shows that post‐operative health is higher in the presence of a bonus, $h^{*}\left(p>0\right)$, than in the absence of a bonus, $h^{*}\left(p=0\right)=\underline{h}+\alpha /c$.^9^

### 2.2 The Purchaser Perspective

We discuss three different approaches the purchaser might adopt in setting up the bonus scheme.

#### 2.2.1 Positive Analysis

First, suppose that the purchaser would like to achieve a health objective, given by $\hat{h}$. A higher bonus p induces a higher level of post‐operative health, while a higher DRG tariff t does not affect health. Because we assume that the number of patients is unaffected by h, the DRG tariff acts as a lump‐sum payment, which is independent of the care provided. The price that implements the objective set by the purchaser is found by equating provider's choice of post‐operative health with purchaser's health objective, $h^{*}\left(p\right)=\hat{h}$:

(8) $$p^{*}\left(\hat{h}\right)=c\left(\hat{h}-\underline{h}\right)-\alpha .$$

Thus, the bonus should be set equal to the marginal cost of post‐operative health at the required target level of health, net of the degree of provider motivation (in line with previous literature, Ellis and McGuire 1986; Chalkley and Malcomson 1998; Kristensen et al. 2016). The greater the degree of motivation, the smaller is the price required to induce the provider to achieve the target health objective.

We can also re‐write the incentive p as a function of post‐operative health in the absence of a bonus system (e.g., before the scheme is implemented or the status quo). In the absence of the scheme, post‐operative health is $h^{*}\left(p=0\right)=\underline{h}+\alpha /c$, and hence

(9) $$p^{*}\left(\hat{h}\right)=c\left(\hat{h}-h^{*}\left(p=0\right)\right).$$

**FIGURE 2:**
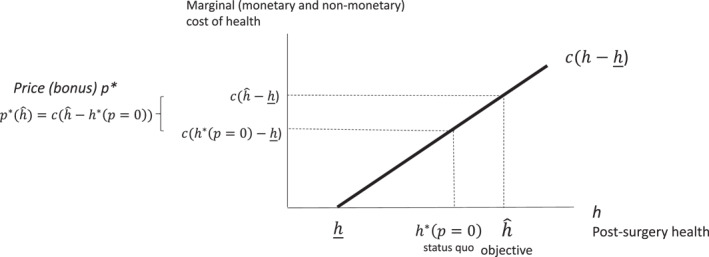
Price designed by the funder to achieve a given health objective.

This is our main result, which will be used in the calibration of the payment system in Section 3. It suggests that the price should be set equal to the difference in the provider marginal cost evaluated at the target level of post‐operative health that the purchaser would like to achieve, $c\left(\hat{h}-\underline{h}\right)$, and the marginal cost evaluated at the level of health before the scheme is introduced, $c\left(h^{*}\left(p=0\right)-\underline{h}\right)$. Figure 2 illustrates the solution. We plot post‐operative health on the x‐axis, and the marginal cost of post‐operative health on the y‐axis. We then evaluate the marginal cost of post‐operative health before the policy intervention (status quo) and after the policy intervention (at the health target). The price (bonus) per unit of post‐operative health is the difference in these two marginal costs.

Provider cost at the new equilibrium following the introduction of the bonus is:

(10) $$\begin{array}{ll} C\left(\hat{h}\right) & =k+\frac{c}{2}{\left(\hat{h}-\underline{h}\right)}^{2}.\; \end{array}$$

Provider costs are higher at the health target $\hat{h}$, and the purchaser has to take these additional costs into account. We have assumed that the purchaser has to ensure that provider costs are covered by provider revenues. Part of the costs are already covered through the bonus payment. The purchaser can then set the HRG tariff equal to the difference between provider costs and provider revenues from the bonus:

(11) $$\hat{t}=C\left(\hat{h}\right)-p^{*}\left(\hat{h}\right)\left(\hat{h}-\tilde{h}\right)=k-c\left[\frac{\hat{h}+\underline{h}}{2}-h^{*}\left(p=0\right)\right]\left(\hat{h}-\tilde{h}\right).$$

**FIGURE 3:**
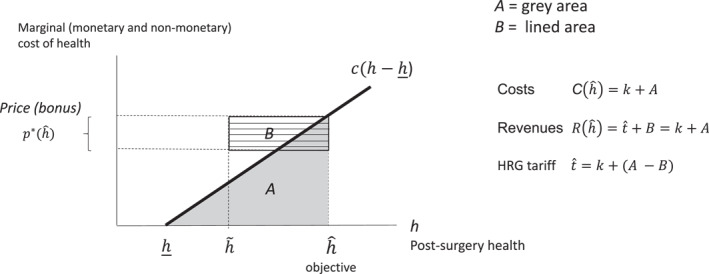
Revenues, costs and basic (HRG) tariff.

Figure 3 illustrates the solution, showing revenues, costs and the HRG tariff for a given health objective $\hat{h}$ that ensures zero profits. Provider variable costs are given by the gray area A. Total costs are given by the fixed cost k plus the variable costs A. The revenues from the bonus are given by area B. Given that total revenues have to be equal to provider costs, the HRG tariff is given by k+(A−B), the fixed cost minus the difference between the variable costs and the revenues from the bonus. In summary, provider revenues from the bonus and the HRG tariff cover hospital costs when the provider chooses a post‐operative health that is equal to the purchaser health target.

#### 2.2.2 Cost‐Effectiveness Analysis

In this section, we provide a framework to assess when the introduction of a P4P scheme that rewards health is cost effective. This approach is relevant in budget‐constrained health systems to evaluate whether the extra health gain is worth the cost of achieving it. As shown in the previous section, provider costs increase as a result of the new health target $\hat{h}$. The difference in provider costs before and after the introduction of the bonus is given by:

(12) $$\Delta C=C\left(\hat{h}\right)-C\left(h^{*}\left(p=0\right)\right)=c\left(\hat{h}-h^{*}\left(p=0\right)\right)\left(\frac{\hat{h}+h^{*}\left(p=0\right)}{2}-\underline{h}\right).$$

Since the purchaser sets the payment such that provider revenues are equal to provider costs, the change in purchaser payment is identical to the change in provider costs.

We define H(h) as patient life‐time gain in health, for example measured in QALYs, arising from a post‐operative health equal to h. The difference in health benefit with and without the bonus is:

(13) $$\Delta H=H\left(\hat{h}\right)-H\left(h^{*}\left(p=0\right)\right).$$

The cost‐effectiveness ratio is:

(14) $$\frac{\Delta C}{\Delta H}=\frac{C\left(\hat{h}\right)-C\left(h^{*}\left(p=0\right)\right)}{H\left(\hat{h}\right)-H\left(h^{*}\left(p=0\right)\right)}.$$

New treatments or interventions are assessed against a threshold value λ for the cost‐effectiveness ratio. The introduction of the bonus payment is cost‐effective if the cost‐effectiveness ratio is below the threshold, ΔC/ΔH<λ and is not cost‐effective if above the threshold, ΔC/ΔH≥λ.

#### 2.2.3 Normative Analysis

In this section, we adopt a conventional welfare economics perspective where a purchaser makes choices that maximize patient benefits, net of provider costs, where the latter are weighted by the opportunity costs of public funds. We also relate this approach to the cost‐effectiveness analysis outlined in the previous section. Define Ω(h) as welfare, δ as the opportunity cost of public funds (or the opportunity cost of the overall health budget) and W as the willingness to pay for health improvement. Welfare is then given by:

(15) Ω(h)=WH(h)−(1+δ)C(h).

The optimal level of post‐operative health satisfies the first‐order condition ∂Ω(h)/∂h=0, or more extensively:

(16) $$W\;\frac{\partial H\left(h\right)}{\partial h}=\left(1+\delta \right)c\left(h-\underline{h}\right),$$

so that, at the optimal level of post‐operative health, the marginal benefit from health gain is equal to the marginal cost. The closed‐form solution for the welfare‐maximizing level of health is

(17) $$h^{w}=\underline{h}+\frac{1}{c}\frac{W}{\left(1+\delta \right)}\frac{\partial H\left(h^{w}\right)}{\partial h}.$$

Comparing the welfare‐maximizing level of health, $h^{w}$, with that chosen by the provider, $h^{*}\left(p\right)$, we can compute the bonus price that implements the welfare‐maximizing level of health^10^ ^:^

(18) $$p^{*}\left(h^{w}\right)=\frac{W}{\left(1+\delta \right)}\frac{\partial H\left(h^{w}\right)}{\partial h}-c\left(h^{*}\left(p=0\right)-\underline{h}\right).$$

The optimal price is equal to the marginal benefit from a health gain, weighted by the opportunity cost of public funds, net of provider marginal cost of health before the introduction of the bonus.

We can also relate this analysis with the cost‐effectiveness approach presented in the previous sub‐section. For simplicity, set $\lambda =W/\left(1+\delta \right)$, and re‐define welfare as λ×H(h)−C(h), which within the cost‐effectiveness literature is known as the net monetary benefit (Stinnett and Mullahy 1998; Hoch et al. 2002). We can re‐write the optimal post‐operative health as $h^{w}=\underline{h}+\frac{\lambda }{c}\frac{\partial H\left(h^{w}\right)}{\partial h}$. Starting from a level of post‐operative health below $h^{w}$, then any improvement in health is cost‐effective as $\lambda \times \frac{\partial H\left(h\right)}{\partial h}-\frac{\partial C\left(h\right)}{\partial h}>0$ or, equivalently,

(19) $$\frac{\partial C\left(h\right)/\partial h}{\partial H\left(h\right)/\partial h}<\lambda ,$$

and any improvement above $h^{w}$ is not cost‐effective as $\frac{\partial C\left(h\right)/\partial h}{\partial H\left(h\right)/\partial h}\geq \lambda$.

## 3 Calibration

To illustrate how the bonus scheme might work in practice, we calibrate the model using English hospital data for two common elective procedures. We use cost and health data for patients receiving a primary unilateral elective hip or knee replacement in 2016–2017. To measure hospital costs, we employ reference costs reported annually by hospitals at the Healthcare Resource Group level, the English equivalent to DRGs.^11^

For each hospital we also measure risk‐adjusted patient health status 6 months after the surgery.^12^ We use the Patient Reported Outcomes Measure dataset constructed from patient survey responses.^13^ We have two measures of patient‐reported health status for each procedure. The first is a condition‐specific measure known as the Oxford Hip Score (OHS) for hip replacement and Oxford Knee Score (OKS) for knee replacement. These measures different dimensions of pain and mobility, and is based on 12 questions giving a score ranging from 0 to 48, with higher values indicating better states of health. The second health measure is the generic EQ‐5D instrument, which asks patient to report whether they have no, some or extreme problems on five health dimensions: mobility, pain & discomfort, anxiety & depression, ability to wash and dress themselves, and ability to carry out their usual activities. The answers form a health profile for which utility values have been elicited from the UK general population. EQ‐5D utility values range from −0.594 to 1. They can be used to calculate quality‐adjusted life years (QALYs). Descriptive statistics are reported in Table 1.

**TABLE 1:** Descriptive statistics. 2016/17.

	Mean	sd	min	max	*N*
Hip replacement					
Cost, MFF adjusted (£)	5859.08	1372.57	1659.96	11,293.33	128
Risk‐adjusted post‐operative health (OHS)	39.42	1.13	34.41	42.07	128
Pre‐operative health (OHS)	17.06	1.66	12.05	21.29	128
Risk‐adjusted health gain (OHS)	21.44	1.13	16.43	24.08	128
Risk‐adjusted post‐operative health (EQ‐5D)	0.79	0.03	0.67	0.85	128
Pre‐operative health (EQ‐5D)	0.32	0.06	0.14	0.45	128
Risk‐adjusted health gain (EQ‐5D)	0.43	0.03	0.31	0.50	128
Knee replacement					
MFF adjusted total cost (£)	5798.55	1231.76	2386.37	9827.19	128
Risk‐adjusted post‐operative health (OHS)	35.58	1.04	31.54	38.21	128
Pre‐operative health (OHS)	18.41	1.49	13.64	22.27	128
Risk‐adjusted health gain (OHS)	16.38	1.04	12.33	19.00	128
Risk‐adjusted post‐operative health (EQ‐5D)	0.74	0.02	0.67	0.81	128
Pre‐operative health (EQ‐5D)	0.39	0.06	0.20	0.52	128
Risk‐adjusted health gain (EQ‐5D)	0.32	0.02	0.25	0.39	128

*Note:* risk‐adjusted post‐operative health gain for provider *i* is equal to: (risk‐adjusted post‐operative health for provider i)—(pre‐operative health averaged across all providers in England).

### 3.1 Hip Replacement

The average cost for a hip replacement across 128 public hospitals (Trusts) in England in 2016–2017 was £5859. The average risk‐adjusted post‐operative health was 39.42 as measured by the Oxford Hip Score. ^14^

We have to make several assumptions to recover the marginal cost parameter *c*, which can be interpreted as the difference in the marginal cost for one unit of health improvement.^15^ Although we cannot observe the marginal cost, we can gather information on some fixed cost components from the literature.^16^ In the baseline calibration (column I of Table 2), we assume that 80% of the costs are fixed, which implies that variable costs account for £1172. There remains uncertainty around the variable costs. Table 1 suggests that the standard deviation of the total provider costs is £1372 (23.4% of the mean). We explore the sensitivity of the results to both lower and higher percentages (70% and 90%). We also need to make assumptions about the value of post‐operative health above which it is costly to increase health, defined with $\underline{h}$ in Section 2, which is another source of uncertainty related to marginal costs. We assume that $\underline{h}$ is equal to 34 OHS, which is just below the minimum post‐operative health observed in the hospital sample (34.41 OHS). We explore below the sensitivity of the results to lower or higher values. Given that $c\left(h^{*}\left(p=0\right)\right)=\frac{c}{2}{\left(h^{*}\left(p=0\right)-\underline{h}\right)}^{2}$, we can re‐cover the cost parameter $c=2\frac{c\left(h^{*}\left(p=0\right)\right)}{{\left(h^{*}\left(p=0\right)-\underline{h}\right)}^{2}}=2\frac{1172}{{\left(39.42-34\right)}^{2}}=£79.8$.

**TABLE 2:** Hip replacement. Calibration under different assumptions on provider costs and purchaser's health objective.

	I	II	III	IV	V	VI	VII	VIII	IX
Observed costs and health									
Hospital cost (£)	5859	5859	5859	5859	5859	5859	5859	5859	5859
$h^{*}\left(p=0\right)$, OHS	39.42	39.42	39.42	39.42	39.42	39.42	39.42	39.42	39.42
*Assumptions*									
% Fixed costs	80%	80%	80%	80%	80%	**70%**	**90%**	80%	80%
Fixed costs (£)	4687	4687	4687	4687	4687	4101	5273	4687	4687
Variable costs (£)	1172	1172	1172	1172	1172	1758	586	1172	1172
$\underline{h}$ (OHS)	34.00	34.00	34.00	34.00	34.00	34.00	34.00	**36.00**	**37.00**
Cost function parameter c (£)	79.8	79.8	79.8	79.8	79.8	119.7	39.9	200.4	400.2
Objective of the funder $\hat{h}$, OHS	40.55	40.55	40.55	**41.12**	**41.68**	40.55	40.55	40.55	40.55
Price p per health improvement (£)	90.1	90.1	90.1	135.2	180.3	135.2	45.1	226.4	452.2
Additional costs									
$C\left(\hat{h}\right)$ (£)	6399	6399	6399	6707	7040	6668	6129	6761	7209
ΔC (£)	540	540	540	848	1181	809	270	902	1350
Revenues									
$\tilde{h}$ (OHS)	38.29	**36.03**	**42.81**	38.29	38.29	38.29	38.29	38.29	38.29
$p*\hat{h}$ (£)	3656	3656	3656	5560	7515	5483	1828	9181	18,337
$p*\left(\hat{h}-\tilde{h}\right)$ (£)	204	407	−204	382	611	306	102	512	1022
HRG tariff $\hat{t}$ (£)	6195	5991	6602	6325	6429	6363	6027	6250	6187

*Note:* $h^{*}\left(p=0\right)$ is provider equilibrium post‐surgery health with no bonus; $\underline{h}$ is the level of health over which improving health is costly for the provider; $\tilde{h}$ is the threshold level of health over which the purchaser reimburses the bonus payment; $\hat{h}$ purchaser health objective; $C\left(\hat{h}\right)$ is the cost of treating the patient at the target health objective; ΔC is the difference in provider cost before and after the introduction of the bonus. Scenario I: baseline; Scenarios II and III: lower and higher threshold over which the purchaser reimburses the bonus; Scenarios IV and V: more ambitious objective of the funder; Scenarios VI and VII: lower and higher proportion of fixed costs (relative to baseline); Scenarios VIII and IX: lower and higher level of health over which improving health is costly for the provider. The text in bold highlights the change in the key assumptions relative to the baseline model.

Abbreviation: OHS = Oxford Hip Score.

In our baseline, we assume that the purchaser sets the objective of the scheme at a post‐operative health, $\hat{h}$, equal to 40.55 OHS. This corresponds to an improvement of 1.13 OHS which is one standard deviation in post‐operative health across 128 providers in 2016–2017 (with a maximum of 42.07 OHS). Using (23), the price per unit of health improvement (one OHS) to achieve the objective $\hat{h}=40.55$ OHS is equal to $p^{*}\left(\hat{h}\right)=c\left(\hat{h}-h^{*}\left(p=0\right)\right)=£90.1$. The cost of a hip replacement at the higher post‐operative health is $C\left(\hat{h}\right)=£6399$, and the additional cost relative to pre‐policy intervention is $\Delta C=C\left(\hat{h}\right)-C\left(h^{*}\left(p=0\right)\right)=£540$.

In terms of provider revenues, the purchaser must decide the level of post‐operative health $\tilde{h}$ (see (1)) over which the purchaser pays for additional health improvements. We set this level at $\tilde{h}=38.29$, which is equal to the pre‐policy post‐operative health *minus* one standard deviation in post‐operative health (39.42−1.13). This threshold is somewhat arbitrary. We have assumed that any additional provider revenues arising from the bonus trigger a reduction in the HRG tariff. In this specific case, the additional revenues from the bonus are $p^{*}\left(\hat{h}\right)\times \left(\hat{h}-\tilde{h}\right)=90.1\times \left(40.55-38.29\right)=£204$. Given that the total cost of a hip replacement is £6399, the basic HRG tariff to ensure that the provider breaks even is equal to $t\left(\hat{h}\right)=£6195$.

The purchaser could instead choose the lower post‐operative health threshold $\tilde{h}=36.06$ (three standard deviations in post‐operative health). Column II of Table 2 suggests that the optimal price remains unchanged given that the health objective is the same. However, the provider receives larger revenues from the bonus, equal to $p^{*}\left(\hat{h}\right)\times \left(\hat{h}-\tilde{h}\right)=90.1\times \left(40.55-36.06\right)=£407$, and these are accompanied by a lower basic HRG tariff equal to £5991, which ensures again that total revenues are equal to provider costs (£6399).^17^

#### 3.1.1 A More Ambitious Target

In columns IV and V of Table 2, we assume that the purchaser sets a more ambitious target with a post‐operative health which is respectively 1.5 and 2 standard deviations higher (rather than one). If the other assumptions remain the same, this implies a price for unit of OHS improvement now equal to £135.2 and £180.3, respectively.

In the first (second) scenario, costs increase to £6707(£7040), which implies an additional cost of £848 (£1181), relative to the introduction of the scheme. Under the same assumptions as in column I in Table 2, the revenues from the bonus are now £382 (£611), which implies that the purchaser reduces the basic HRG tariff to £6325 (£6429) to ensure that the costs are covered.

As intuitively expected, the price per unit of health improvement increases with the ambition of the target. The higher level of health increases costs and revenues, and the purchaser therefore needs to adjust the basic HRG tariff to reflect the higher level of the cost.

#### 3.1.2 Different Cost Function

We have assumed that 80% of costs are fixed. In columns VI and VII we make the same assumptions as in column I but change the proportion of costs that are fixed to 70% and 90%, respectively. The price per unit of health improvement now increases (reduces) to £135.2(£45.1). The cost parameter c also increases (reduces) to £119.7 (£39.9). Driven by the higher tariff, the additional revenues from the bonus also increase (reduce). The basic HRG tariff also increases (reduces), despite the higher bonus, and this is driven by the higher (lower) additional costs ΔC=£809(£270).

We can also vary the minimum level of post‐operative health over which costs increase, which we have set at $\underline{h}=34$ OHS. In columns VIII and IX, we maintain the same assumptions as in column I but increase $\underline{h}$ to 36 and 37, respectively. This implies an increase in the cost function parameter c to £200.4 and £400.2, which translate into a higher price per unit of health improvement equal to £226.4 and £452.2. The higher marginal cost of a health improvement implies that costs at the new health target are £6761 and £7209, respectively. The revenues from the bonus are now £512 and £1022, with the basic HRG tariff being re‐adjusted to cover the costs.

#### 3.1.3 Cost‐Effectiveness Analysis

To investigate whether the introduction of the incentive scheme is cost‐effective, we set the health objective of the purchaser as a function of EQ‐5D utility scores. Following similar steps as above, column I of Table 3 provides our baseline. The pre‐policy post‐operative health now gives a EQ‐5D utility score of 0.79 and the purchaser objective is 0.82.^18^ To simplify the presentation, we measure post‐operative health with the EQ‐5D utility score multiplied by 100, so that a post‐operative health of 0.79 is measured as h=79.

**TABLE 3:** Hip replacement. Cost‐effectiveness.

	I	II	III	IV	V	VI	VII
Observed costs and health							
Cost (£)	5859	5859	5859	5859	5859	5859	5859
$h^{*}\left(p=0\right)$, EQ‐5D (out of 100)	79.09	79.09	79.09	79.09	79.09	79.09	79.09
Assumptions							
% Fixed costs	80%	80%	80%	**70%**	70%	70%	70%
Fixed costs	4687	4687	4687	4101	4101	4101	4101
Variable costs	1172	1172	1172	1758	1758	1758	1758
$\underline{h}$	66.65	**69.55**	**72.45**	72.45	72.45	72.45	72.45
Cost function parameter c (£)	15.1	25.8	53.2	79.7	79.7	79.7	79.7
Objective of the funder $\hat{h}$	81.99	81.99	81.99	81.99	**84.89**	84.89	**91.84**
Price p per health improvement (£)	43.9	74.7	154.2	231.2	462.5	462.5	1016.2
Additional costs							
$C\left(\hat{h}\right)$ (£)	6469	6680	7106	7730	10,271	10,271	19,082
ΔC (£)	610	821	1247	1871	4412	4412	13,223
Revenues							
$\tilde{h}$	76.19	76.19	76.19	76.19	76.19	76.19	76.19
$p*\hat{h}$ (£)	3601	6123	12,639	18,958	39,258	39,258	93,323
$p*\left(\hat{h}-\tilde{h}\right)$ (£)	255	433	894	1341	4023	4023	15,898
HRG tariff $\hat{t}$ (£)	6214	6247	6212	6389	6247	6247	3184
Cost effectiveness							
QALY improvement for 0.01 EQ‐5D	0.1149	0.1149	0.1149	0.1149	0.1149	**0.1030**	**0.1030**
QALY improvement	0.3332	0.3332	0.3332	0.3332	0.6664	0.5974	1.3133
Cost per QALY	1831	2463	3743	5614	6620	7385	10,069

*Note:* $h^{*}\left(p=0\right)$ is provider equilibrium post‐surgery health with no bonus; $\underline{h}$ is the level of health over which improving health is costly for the provider; $\tilde{h}$ is the threshold level of health over which the purchaser reimburses the bonus payment; $\hat{h}$ purchaser health objective; $C\left(\hat{h}\right)$ is the cost of treating the patient at the target health objective; ΔC is the difference in provider cost before and after the introduction of the bonus. The text in bold highlights the change in the key assumptions relative to the baseline model.

Under the assumption that 80% of the costs are fixed, and $\underline{h}=66.65$ (i.e., 0.6665 EQ‐5D utility score, about the minimum level observed in the hospital sample), then the optimal price is £43.9 per unit of health improvement (0.01 EQ‐5D utility score). With a payment health threshold of 76 (0.76 EQ‐5D utility score), the hospital revenues from the bonus are £255, and the basic HRG tariff is £6214, with an overall provider cost of £6469.

We assume that the health gain over the patient life span for a 0.01 EQ‐5D utility score improvement is 0.1149 QALYs (ranging from 0.103 under more conservative estimates to 0.126), computed as follows. We follow Briggs et al. (2004) and Jenkins et al. (2013) and draw from the expected remaining years of life of the general population from Office for National Statistics Life Tables, and use the expected life of patients with the average age of our empirical sample in 2016/17, amounting to 18 years. We then adjust for the mortality risk in each of these years.^19^ Finally, we discount future health gains using a time preference discount rate ranging from 1.5% (Claxton et al. 2011) to 3.5% as recommended by HM Treasury. Under these different assumptions the QALY improvement from hip replacement ranges from 0.103 to 0.126 QALYs (see also Appleby et al. 2013; Schmitz et al. 2019).

In terms of cost effectiveness, the incremental cost per QALY is £1831, suggesting that the introduction of the scheme is cost effective, well below the conventional thresholds used in economic evaluation of healthcare programmes (Claxton et al. 2015; Lomas et al. 2019).

In column II‐VI we investigate whether this result is robust to less favorable assumptions of the cost function. In columns II and III of Table 3, we increase $\underline{h}$ to higher values, which increase the cost parameter c from £15.1 to £25.8 and £53.2, leading to the higher prices of £74.7 and £154.2. In column IV we further increase the proportion of variable costs to 30%, which implies a price of £231.2. In column V we maintain the assumptions in column IV but set the health target at 84.89, equivalent to two standard deviation improvement. The price is 462.5 per unit of health improvement, revenues from the bonus are £4023, and an overall provider cost of £10271. Despite the very high costs (relative to pre‐policy level of £5859), the introduction of the bonus remains cost‐effective with a cost per QALY of £6620. Column VI uses the more conservative estimate of a QALY gain of 0.103 for a 0.01 EQ‐5D utility score improvement. The cost per QALY is £7385.

This analysis suggests that for the purchaser it is generally cost effective to set an ambitious health target.

#### 3.1.4 Normative Analysis

Based on Section 2.2.3, we can compute the optimal level of post‐operative health which equates purchaser's marginal benefit with its marginal cost, given by $h^{w}=\underline{h}+\frac{1}{c}\frac{W}{\left(1+\delta \right)}\frac{\partial H\left(h^{w}\right)}{\partial h}$. We choose the most conservative parameters with highest costs and lowest benefits in column VI of Table 3: 30% variable costs and $\underline{h}=72.45$, leading to c=£79.7; a QALY improvement from an increase in one unit of post‐operative health equal to 0.1030, so that $\frac{\partial H\left(h^{w}\right)}{\partial h}=0.1030$; and a willingness to pay for a QALY equal to $\frac{W}{\left(1+\delta \right)}=£15000$.

By substitution, we obtain an optimal level of post‐operative health equal to $h^{w}=91.8$. This is equivalent to an improvement of more than four standard deviations in post‐operative health relative to the pre‐policy level, well above the maximum post‐operative health observed across providers equal to 85 (0.85 EQ‐5D).

Recall that the optimal pricing rule is $p^{*}\left(h^{w}\right)=\frac{W}{\left(1+\delta \right)}\frac{\partial H\left(h^{w}\right)}{\partial h}-c\left(h^{*}\left(p=0\right)-\underline{h}\right)$. The optimal price which implements the level of post‐operative health $h^{w}=91.8$ is then equal to $p^{*}\left(h^{w}\right)=15000*\left(0.1030\right)\;-\left(79.7\right)\left(79.09-72.45\right)=£1016$. The results are in column VII of Table 3. The cost of a hip replacement at such high levels of post‐operative health is about three times the cost at the baseline, £19082. Most of hospital revenues arise from the bonus, £15898, and the remaining from the HRG tariff, £3184. The incremental cost per QALY relative to the baseline (given by $\frac{\Delta C}{\Delta H}=\frac{C\left(\hat{h}\right)-C\left(h^{*}\left(p=0\right)\right)}{H\left(\hat{h}\right)-H\left(h^{*}\left(p=0\right)\right)}$) is further increased, and equal to £10069. The marginal cost‐effectiveness ratio evaluated at the optimal post‐operative health is instead $\frac{\partial C\left(h^{w}\right)/\partial h}{\partial H\left(h^{w}\right)/\partial h}=\frac{c\left(h^{w}-\underline{h}\right)}{0.1030}=\frac{79.7\left(91.8-72.45\right)}{0.1030}\approx 15000=\frac{W}{\left(1+\delta \right)}$, which is higher than the incremental cost‐effectivness ratio. The result that the marginal cost‐effectiveness ratio is equal to the purchaser willingness to pay holds by construction because the optimal price is computed such that the marginal benefit equates its cost.

We have chosen conservative measures of parameter values. Both higher benefits or lower costs would increase further the optimal post‐operative health set by the purchaser.

### 3.2 Knee Replacement

We replicate the analysis for knee replacement. The findings are qualitatively and quantitatively similar to those obtained for hip replacement. The price for one unit of health improvement as measured by the Oxford Knee Score (OKS) to achieve an improvement of 1.06 points on the OKS scale (one standard deviation) ranges between £72 and £254 under different assumptions on costs. See Appendix for details.

## 4 Heterogeneity Across Providers

In this section, we extend the analysis to a set‐up where hospitals differ in the marginal cost of improving health. We assume that the purchaser uses the same health‐related bonus p and DRG tariff t, as described in (1), for all providers. We assume that the cost function is:

(20) $$C\left(h,e\right)=k+\frac{c}{2e}{\left(h-\underline{h}\right)}^{2},$$

where e is a parameter denoting provider type and is distributed with cumulative function F(e) and density function f(e) over the support $\left[e_{\min},e_{\max}\right]$ with an expected value normalized to one, $E\left(e\right)=\int_{e_{\min}}^{e_{\max}}ef\left(e\right)de=1$. For a given bonus payment, the optimal level of post‐operative health by each provider is $h^{*}\left(p,e\right)=\underline{h}+\frac{\left(p+\alpha \right)e}{c}$. We assume that $e_{\max}$ is the most efficient provider (lowest marginal cost) and $e_{\min}$ is the least efficient provider (has highest marginal cost), with $e_{\max}>1$ and $0<e_{\min}<1$.

### 4.1 Positive Analysis

Suppose that the purchaser would like to achieve an average health across providers equal to $\hat{h}=E\left(h^{*}\left(p,e\right)\right)$. The bonus payment that implements this target health is such that

(21) $$\int_{e_{\min}}^{e_{\max}}\left(\underline{h}+\frac{\left(p+\alpha \right)e}{c}\right)f\left(e\right)de=\hat{h},$$

which is given by:

(22) $$\hat{p}=p^{*}\left(\hat{h}\right)=c\left(\hat{h}-\underline{h}\right)-\alpha ,$$

where recall that E(e)=1 by assumption. The optimal bonus per unit of health improvement is the same as in (8). Given that, in the absence of a bonus, the expected health is equal to $E\left(h^{*}\left(p=0,e\right)\right)=\int_{e_{\min}}^{e_{\max}}\left(\underline{h}+\frac{\alpha e}{c}\right)f\left(e\right)de=\underline{h}+\frac{\alpha }{c}$, we can re‐write this bonus payment as a function of post‐operative health in the absence of a bonus (e.g., before the scheme is implemented):

(23) $$\hat{p}=c\left(\hat{h}-E\left(h^{*}\left(p=0\right)\right)\right).$$

This result is qualitatively similar to Section 2 with a representative provider.

We assume that the DRG tariff $\hat{t}$ is set such that the expected revenues across providers are equal to the expected costs. This is in line with DRG‐type reimbursement systems that set the DRG tariff equal to the average cost across providers. In turn, this implies that some providers will make a profit while others will make a loss.

The cost for provider e when the bonus payment is set at $\hat{p}$, after substituting $h^{*}\left(\hat{p},e\right)=\underline{h}+\left(\hat{h}-\underline{h}\right)e$, is equal to

(24) $$C\left(h^{*}\left(\hat{p},e\right),e\right)=k+\frac{c}{2}{\left(\hat{h}-\underline{h}\right)}^{2}e,$$

and the expected cost is equal to

(25) $$E\left(C\left(h^{*}\left(\hat{p},e\right),e\right)\right)=k+\frac{c}{2}{\left(\hat{h}-\underline{h}\right)}^{2}.$$

Similarly, the revenues for provider e for a given DRG tariff t is equal to: $R\left(\hat{p},e\right)=t+\hat{p}\left(h^{*}\left(\hat{p},e\right)-\tilde{h}\right)$. More efficient providers have higher post‐operative health and revenues from the bonus payment. The expected revenue across providers is $E\left(R\left(\hat{p},e\right)\right)=t+\hat{p}\left(\hat{h}-\tilde{h}\right)$. The purchaser can then compute the DRG tariff that ensures that the expected revenues are equal to the expected costs across all providers:

(26) $$\hat{t}=k+\frac{c}{2}{\left(\hat{h}-\underline{h}\right)}^{2}-\hat{p}\left(\hat{h}-\tilde{h}\right).$$

More efficient providers have higher revenues but also higher costs. Whether more efficient providers have a higher financial surplus depends on:

(27) $$\frac{d\left(R\left(\hat{p},e\right)-C\left(\hat{p},e\right)\right)}{de}=\left(\frac{c}{2}\left(\hat{h}-\underline{h}\right)-\alpha \right)\left(\hat{h}-\underline{h}\right),$$

which suggests that financial surplus increases with efficiency only if the degree of altruism is sufficiently low.

### 4.2 Cost‐Effectiveness Analysis

Given the target $\hat{h}$, the difference in provider expected costs before and after the introduction of the bonus is given by:

(28) $$\Delta E\left(C\right)=E\left(C\left(h^{*}\left(\hat{p},e\right),e\right)\right)-E\left(C\left(h^{*}\left(p=0,e\right),e\right)\right).$$

Similarly, the difference in expected health benefit with and without the bonus is:

(29) $$\Delta E\left(H\right)=E\left(H\left(h^{*}\left(\hat{p},e\right)\right)\right)-E\left(H\left(h^{*}\left(p=0,e\right)\right)\right),$$

where

(30) $$E\left(H\left(h^{*}\left(p,e\right)\right)\right)=\int_{e_{\min}}^{e_{\max}}H\left(h^{*}\left(p,e\right)\right)f\left(e\right)de.$$

The cost‐effectiveness ratio is then given by ΔE(C)/ΔE(H). Policy interventions are assessed against a threshold value for the cost‐effectiveness ratio. Letting λ denote this cost‐effectiveness threshold then the bonus is cost‐effective if ΔE(C)/ΔE(H)<λ and is not cost‐effective if instead ΔE(C)/ΔE(H)≥λ. The results are qualitatively similar to those derived in Section 2.2.2.

### 4.3 Normative Analysis

The welfare function is given by the difference between patient's benefits and the transfer to providers, weighted by the opportunity cost of public funds δ:

(31) $$\Omega \left(h\right)=\int_{e_{\min}}^{e_{\max}}\left\{W\;H\left(h^{*}\left(p,e\right)\right)-\left(1+\delta \right)\left[t+p\left(h^{*}\left(p,e\right)-\tilde{h}\right)\right]\right\}f\left(e\right)de.$$

Welfare is maximized subject to the budget constraint that the expected revenues are equal to the expected costs across providers:

(32) $$\int_{e_{\min}}^{e_{\max}}\left[t+p\left(h^{*}\left(p,e\right)-\tilde{h}\right)-C\left(h^{*}\left(p,e\right),e\right)\right]f\left(e\right)de\geq 0.$$

Substituting for the budget constraint, the optimal payment per unit of health improvement p is such that it satisfies the following first‐order condition:

(33) $$\int_{e_{\min}}^{e_{\max}}\left\{W\;\frac{\partial H\left(h^{*}\left(p,e\right)\right)}{\partial h}-\left(1+\delta \right)\frac{\partial C\left(h^{*}\left(p,e\right),e\right)}{\partial h}\right\}\frac{\partial h^{*}\left(p,e\right)}{\partial p}f\left(e\right)de=0.$$

Substituting $\frac{\partial C\left(h^{*}\left(p,e\right),e\right)}{\partial h}=\alpha +p$ for any e, we can write the optimal payment per unit of health gain, denoted with $p^{w}$ as:

(34) $$p^{w}=\frac{W}{\left(1+\delta \right)}\int_{e_{\min}}^{e_{\max}}\frac{\partial H\left(h^{*}\left(p^{w},e\right)\right)}{\partial h}ef\left(e\right)de-\alpha .$$

The optimal price is equal to the expected marginal benefit from a health gain, weighted by the opportunity cost of public funds, net of provider degree of motivation. The optimal pricing rule is analogous to the one obtained in Section 2.2.3, except that the marginal benefit is averaged across all providers. We can also re‐write the pricing rule as:

(35) $$p^{w}=\frac{W}{\left(1+\delta \right)}\int_{e_{\min}}^{e_{\max}}\frac{\partial H\left(h^{*}\left(p^{w},e\right)\right)}{\partial h}ef\left(e\right)de-\int_{e_{\min}}^{e_{\max}}\frac{\partial C\left(h^{*}\left(p=0,e\right),e\right)}{\partial h}ef\left(e\right)de,$$

where the second term can be interpreted as the expected marginal cost across providers in the absence of a bonus, given that $\frac{\partial C\left(h^{*}\left(p=0,e\right),e\right)}{\partial h}=\alpha$. Again, the optimal pricing rule is qualitatively similar to Section 2.2.3 but with marginal benefit and cost averaged across providers.

The purchaser can then compute the optimal DRG tariff $t^{w}$ that ensures that the expected revenues are equal to the expected costs across all providers:

(36) $$t^{w}=\int_{e_{\min}}^{e_{\max}}\left[C\left(h^{*}\left(p^{w},e\right),e\right)-p^{w}\left(h^{*}\left(p^{w},e\right)-\tilde{h}\right)\right]f\left(e\right)de\geq 0.$$

The DRG tariff ensures that *on average* providers do not make losses.

### 4.4 Calibration

Consider the baseline calibration for hip replacement (column I of Table 2). For the positive analysis, we assume that the purchaser's objective is to increase the average post‐operative health from 39.42 to 40.55 on the Oxford Hip Score scale (or one standard deviation in post‐operative health across hospitals). Given that the expected cost is $E\left(C\left(h^{*}\left(p=0,e\right),e\right)\right)=k+\frac{c}{2}\alpha^{2}$, and that $E\left(h^{*}\left(p=0,e\right)\right)=\underline{h}+\frac{\alpha }{c}$, we can re‐write the expected cost as $E\left(C\left(h^{*}\left(p=0,e\right),e\right)\right)=k+\frac{c}{2}{\left[E\left(h^{*}\left(p=0,e\right)\right)-\underline{h}\right]}^{2}$. If we assume again that 80% of the costs are fixed, which implies that variable costs account for £1172, and that the value of post‐operative health above which it is costly to increase health, $\underline{h}$ is equal to 34 OHS, we can re‐cover the cost parameter $c=2\frac{E\left(C\left(h^{*}\left(p=0,e\right),e\right)\right)-k}{{\left.\left[E\left(h^{*}\left(p=0,e\right)\right)-\underline{h}\right]\right)}^{2}}=2\frac{1172}{{\left(39.42-34\right)}^{2}}=£79.8$. The price that per unit of health improvement (one point on the Oxford Hip Score) that implements the purchaser's target is $\hat{p}=c\left(\hat{h}-E\left(h^{*}\left(p=0\right)\right)\right)=79.8\left(40.55-30.42\right)=£90.1$.

To ensure that every provider receives a positive bonus, we set the threshold post‐operative health over which payment is received by the provider at $\tilde{h}=33$ (differently from Section 3.1 where it was set at 38.29). Given that providers differ in their degree of efficiency, they will receive different bonus payments which are proportional to their post‐operative health, $h^{*}\left(\hat{p},e\right)=\left(\hat{h}-\underline{h}\right)e$. We can recover the distribution of efficiency by the observed distribution of post‐operative health before the introduction of the bonus payment: $e=\frac{h^{*}\left(p=0,e\right)-\underline{h}}{E\left(h^{*}\left(p=0,e\right)\right)-\underline{h}}$.^20^ The least efficient provider has the lowest pre‐policy post‐operative health, which implies $e_{\min}=\frac{\left(34.41-34\right)}{\left(39.42-34\right)}=0.076$ while for the most efficient provider $e_{\max}=\frac{\left(42.07-34\right)}{\left(39.42-34\right)}=1.489$.

The post‐operative health, following the introduction of the policy that sets a target equal to $\hat{h}=40.55$, for the least and most efficient provider is $h^{*}\left(\hat{p},e_{\min}\right)=\left(\hat{h}-\underline{h}\right)e_{\min}+\underline{h}=\left(40.55-34\right)0.076+34=34.50$ and $h^{*}\left(\hat{p},e_{\max}\right)=\left(\hat{h}-\underline{h}\right)e_{\max}+\underline{h}=\left(40.55-34\right)1.489+34=43.75$. Therefore, the least efficient provider only increases post‐operative health by 0.09 units on the Oxford Hip Score scale and the most efficient by 1.68 units, with an average health improvement of 1.13 units.

Such differences in post‐operative health also translate into different bonus payments. Given that the bonus payment for provider e is equal to $\hat{p}\left[h^{*}\left(\hat{p},e\right)-\tilde{h}\right]$, the bonus payment for the least and most efficient provider is respectively equal to £137.4 and £968.8, while for the average provider is £680.3. The bonus payment for the average provider is higher than the one obtained in Section 3.1 due to the lower threshold over which the provider is reimbursed.

To determine the HRG tariff, the purchaser has to compute the expected cost, which in line with Section 3.1, is equal to $E\left(C\left(h^{*}\left(\hat{p},e\right),e\right)\right)=k+\frac{c}{2}{\left(\hat{h}-\underline{h}\right)}^{2}=£6399$. The HRG tariff is then equal to. $\hat{t}=E\left(C\left(h^{*}\left(\hat{p},e\right),e\right)\right)-\hat{p}\left(\hat{h}-\tilde{h}\right)=6399-680.3=£5718.7$

In summary, although heterogeneous providers differ in their response to the incentive scheme, the derivation of the price does not differ qualitatively to one where the scheme is set for the average provider.

## 5 Conclusions

The availability of routinely collected health outcome measures raises the appealing possibility of conditioning payment on what really matters to patients, the health gains. One key question is how any outcome‐related bonus should be set. To address this question we analyzed a simple but general model based on contract theory. The model suggests that the price per unit of health improvement should be set equal to the difference between the cost of a unit of health improvement at the target level of health (after the policy has been introduced) and the cost of a unit of health improvement before the policy been introduced. Although higher prices lead to higher provider revenues, the purchaser can reduce the base (DRG/HRG) tariff to ensure that the provider overall revenues (from the HRG tariff plus the bonus) cover provider costs. Higher health targets are more costly for the provider, and therefore the higher costs have to be covered by the purchaser through higher revenues either from the bonus or the HRG tariff. The model also derives the conditions required by the purchaser to implement interventions that are either cost‐effective or welfare maximizing.

The model provides the basis for establishing what level of bonus might be required in practice. We examined data on two procedures in the English NHS where health outcome measures are available—hip and knee replacements. In our calibration for hip replacement, we find that the price per unit of health improvement as measured by the Oxford Hip Score to achieve an improvement of 1.13 OHS (one standard deviation in provider distribution) ranges between £45 and £226 under different assumptions related to the cost function. For knee replacement, the price per unit of health improvement as measured by the Oxford Knee Score to achieve an improvement of 1.06 OKS (one standard deviation) ranges between £72 and £254. The price doubles for a health target of two, rather than one, standard deviation improvement.

When we evaluate the health benefits in QALYs, we generally find that the introduction of the bonus is cost‐effective, even at high levels of health targets. In turn, this implies that under a welfare function approach, the optimal health target is high and the price per unit of health improvement is even higher than those considered in the previous scenarios.^21^

Pay for performance is only one of several mechanisms that purchasers can implement to raise quality. Other mechanisms include regulatory and monitoring mechanisms, such as audits to ensure minimum standards. Similarly, regulators can use quality indicators for public reporting and benchmarking to inform patient choice and and stimulate provider competition on quality. In our model, we assume that providers are motivated, which is the result of altruistic concerns towards patients but also other existing mechanisms that stimulate quality and contribute to the “status quo”, before the P4P policy is being introduced. The main implication is that the stronger are other mechanisms to improve quality, the lower is the scope for P4P and the lower is the bonus payment necessary to induce the target level of health set by the purchaser.

Another policy implication, as shown by our heterogeneity analysis, is that P4P exposes providers to uncertainty in relation to revenues and profits. However, P4P has been introduced only for few procedures. For losses to be a problem, purchasers would have to use P4P across many procedures and, at the same time, some hospitals would have to systematically provide low quality across all procedures leading to losses and deficits.

In terms of limitations, the analysis has abstracted from modeling patient heterogeneity in severity. This is because PROMs are risk‐adjusted, and include the pre‐operative health in the casemix, which is a good proxy of capacity to benefit. Moreover, if the payment scheme is set to improve the health of patients on average, as opposed to an individual patient, any source of unobserved severity will balance out if it is not systematic. We also did not consider heterogeneity between treatments. Future work could investigate the design of P4P in the presence of patient heterogeneity both within and across treatments (e.g., due to patient severity, ability to benefit or socioeconomic status) and incorporate equity concerns in the welfare function (Siciliani and Straume 2019), for example by allowing willingness to pay to vary with severity or health status, or use distributional cost‐effectiveness analysis (Asaria et al. 2016).^22^

Our analysis highlighted the critical role of the marginal cost of health improvements. We had to make several assumptions to recover this parameter. Future work could estimate the marginal cost of health for specific procedures though this would require robust identification strategies to address simultaneity and omitted‐variable biases.

## Conflicts of Interest

The authors declare no conflicts of interest.

## Data Availability

Data sharing not applicable to this article as no datasets were generated or analyzed during the current study.

## Endnotes

^1^
One exception is the Advancing Quality scheme for hospitals in England, which incentivised reductions in mortality for emergency conditions such as acute myocardial infarction, heart failure and pneumonia. This reduced mortality but had spillover effects on non‐incentivised conditions (Britteon et al. 2025).

^2^
One exception in the context of primary care is the Quality and Outcome Framework (QOF) scheme in England where the incentive payment was initially around 20% of general practice income (Gravelle et al. 2010).

^3^
For practical measurement purposes, we can think of patient health h being measured by EQ‐5D utility scores or with a procedure specific metric, such as the Oxford Hip Score.

^4^
We assume that the volume of patients treated is fixed, and do not consider possible indirect effects of the bonus on volume. For example, by inducing an increase in quality, the provider may respond to the bonus by reducing volume due to pressures on costs.

^5^
Suppose for example that the threshold is set at $\tilde{h}=42$ OHS. If post‐operative health is h=38 then hospital payment is $p\left(h-\tilde{h}\right)=100\left(38-42\right)=-£400$, and every additional health improvement reduces such penalty.

^6^
Within the English NHS both doctors and nurses are salaried. Therefore, any additional time spent with the patient will not increase costs unless they are working at full capacity, and the only way to increase time with the patients is by recruiting additional personnel.

^7^
This assumption is consistent with the empirical findings of Gutacker et al. (2013), which shows that at the sample mean higher health gains do not lead to statistically significant higher monetary costs.

^8^
For simplicity we ignore any upper or lower bound on health gain and assume that for the given parameters, the provider's choice lies within a feasible range.

^9^
Given the hospital's choice of post‐operative health $h^{*}\left(p\right)$  the cost of treating a patient, revenues and financial surplus are: $C\left(h^{*}\left(p\right)\right)=k+\frac{{\left(p+\alpha \right)}^{2}}{2c}$, $R\left(h^{*}\left(p\right),p\right)=t+p\left(\frac{p+\alpha }{c}+\underline{h}-\tilde{h}\right)$, and $\pi \left(h^{*}\left(p\right),p\right)=t-k+\frac{p^{2}-\alpha^{2}}{2c}-p\left(\tilde{h}-\underline{h}\right)$. The introduction of a bonus p>0 always increases costs since, from (7) a higher bonus p increases health and greater health is more costly to achieve: $dC\left(h^{*}\left(p\right)\right)/dp$ $=\left(dC/dh\right)dh^{*}/dp$ $=\left(p+\alpha \right)/c$. The bonus also increases revenue: $dR\left(h^{*}\left(p\right),p\right)/dp$ $=\left(\partial R/\partial h\right)dh^{*}/dp$ + $\partial R/\partial p=\left(2p+\alpha \right)/c+\underline{h}-\tilde{h}$. An increase in the bonus always increases the provider utility V and its financial surplus π: $\frac{dV\left(h^{*}\left(p\right),p\right)}{dp}=\frac{\partial V\left(h^{*}\left(p\right),p\right)}{\partial h}\frac{dh^{*}}{dp}+\frac{\partial V\left(h^{*}\left(p\right),p\right)}{\partial p}=\frac{\partial V\left(h^{*}\left(p\right),p\right)}{\partial p}=\frac{\partial \pi \left(h^{*}\left(p\right),p\right)}{\partial p}=h^{*}\left(p\right)-\tilde{h}>0$. This follows because h is chosen to maximise V so that ∂V(h,p)/∂h=0 by the envelop theorem.

^10^
We have $p^{*}\left(h^{w}\right)=\frac{W}{\left(1+\delta \right)}\frac{\partial H\left(h^{w}\right)}{\partial h}-\alpha$. Recalling that $h^{*}\left(p=0\right)=\underline{h}+\alpha /c$, and substituting for α the result is obtained.

^11^
Hospitals report the average cost of care for patients with a length of stay shorter than a HRG‐specific trim point and the average additional cost per day of caring for patients with a longer length of stay than this trim point. Private providers do not report HRG costs and are therefore excluded.

^12^
Case‐mix adjustment for post‐operative health uses an official model created to monitor hospital performance through PROMs. The model includes pre‐operative health, age, gender, ethnicity, deprivation, assistance in completing the PROM form, specific comorbidities, primary diagnosis, disability and history of symptoms. The set of variables used is measure specific, based on significant coefficients from a common set of candidate characteristics (NHS England 2013).

^13^
From April 2009, it has been mandatory for all hospitals to provide patients with these surveys shortly before and six months after undergoing elective (planned) hip and knee replacement. About 48% of patients completed both a pre‐operative and post‐operative survey.

^14^
The average pre‐operative OHS was instead 17.06 across hospitals. The average health gain across providers was instead 21.44 OHS. Notice that the latter is computed for each provider as the risk‐adjusted post‐operative health minus a constant term given by the average pre‐operative health across English hospitals.

^15^
As discussed below, we measure health first on the OHS scale, where one unit of improvement is just below one standard deviation of the post‐operative distribution. We then measure health on a 0–100 scale where 1 unit is equivalent to 0.01 units of health on the EQ‐5D scale as this is again clinically meaningful given that post‐operative health has a standard deviation of 0.03 when measured on the EQ‐5D scale.

^16^
For example, one significant fixed cost of a hip replacement is the cost of a prosthesis, which accounts for between 26% and 53% of the total cost (Mota 2013; Clarke et al. 2015).

^17^
The purchaser could also set up the scheme as a penalty rather than a bonus. For example, the purchaser could penalise each provider with a penalty equal to £90.1 for every unit of OHS that is below the higher threshold of 42.81, which is three standard deviations in post‐operative health above the pre‐policy level (column III in Table 2). Given that the scheme incentivises the provider to reach a post‐operative health equal to 40.55, as for columns I and II, the provider now makes a financial loss of $p^{*}\left(\hat{h}\right)\times \left(\hat{h}-\tilde{h}\right)=-90.1\times \left(42.81-40.55\right)=-£204$. Given that the provider has to break even and cover the costs of £6399, now the HRG tariff is increased by the purchaser to £6602.

^18^
We assume that the health target is specified in post‐operative health, and not in QALYs, as the former is what the purchaser observes 6 months after the surgery. The purchaser can however take into account the QALYs generated by an improvement in post‐operative health when setting the health target.

^19^
To adjust for the mortality risk in each of these years, we use the mortality ratios indicated in past literature between hip replacement patients and the general population over the long term, which range from 0.7 (Pedersen et al. 2011) to 0.95 (Visuri et al. 2010). Short term mortality (usually the first 90 days following a procedure) is not of concern for us as post‐operative health is reported 6 months after the procedure and so is conditional on survival for this period. We further adjust for a risk of revision based on revision rates of different types of hip replacement over 10 years reported in Pennington et al. (2013).

^20^
Given that $E\left(h^{*}\left(p=0,e\right)\right)=\underline{h}+\frac{\alpha }{c}$, substituting for α into $h^{*}\left(p=0\right)=\underline{h}+\frac{\alpha e}{c}$, we obtain the degree of efficiency as a function of the pre‐policy distribution of post‐operative health.

^21^
Whilst we refer to the incentive scheme as a bonus it could also be set up as a penalty for shortfalls from the health target set by the purchaser. Here, the price per health improvement remains critical and can be interpreted as the reduction in the penalty for unit of health improvement. However, if the provider ultimately incurs a loss, the purchaser will have to compensate such financial loss with a higher HRG tariff to ensure that the provider breaks even.

^22^
Similarly, we may be concerned about measurement error in the health measures, which could expose providers to losses or grant them profits. However, hip and knee replacements are common procedures, with most hospitals treating hundreds of patients. Therefore, any measurement error should balance out with large volumes. If risk adjustment is imperfect and there is unaccounted heterogeneity that providers can observe prior to committing to treatment then there is a risk that providers will select patients to treat and potentially avoid difficult patients. This issue does not seem particularly relevant in the system we study where hospitals are mostly obligated to treat those patients who are referred to them. Neither have we considered the issue of multitasking (tunnel vision). This is because our measures of health are comprehensive, therefore any incentive to divert effort from untargeted to targeted care should be minimal.

## Calibration. Knee Replacement

**TABLE A1:** Knee replacement. Calibration under different assumptions on costs and purchaser health objective.

	I	II	III	IV	V	VI	VII	VIII	IX	X
Observed costs and health										
Cost (£)	5799	5799	5799	5799	5799	5799	5799	5799	5799	5799
$h^{*}\left(p=0\right)$, OKS	35.58	35.58	35.58	35.58	35.58	35.58	35.58	35.58	35.58	35.58
Assumptions										
% Fixed costs	80%	80%	80%	80%	80%	**70%**	**90%**	80%	80%	**70%**
Fixed costs (£)	4639	4639	4639	4639	4639	4059	5219	4639	4639	4059
Variable costs (£)	1160	1160	1160	1160	1160	1740	580	1160	1160	1740
$\underline{h}$ (OKS)	31.50	31.50	31.50	31.50	31.50	31.50	31.50	**32.50**	**33.50**	**33.50**
Cost function parameter c (£)	139.3	139.3	139.3	139.3	139.3	209.0	69.7	244.4	535.9	803.8
Objective of the funder $\hat{h}$, OKS	36.62	36.62	36.62	**37.14**	**37.66**	36.62	36.62	36.62	36.62	**37.66**
Price p per health improvement (£)	144.9	144.9	144.9	217.3	289.8	217.3	72.4	254.2	557.3	1672.0
Additional costs										
$C\left(\hat{h}\right)$ (£)	6465	6465	6465	6855	7282	6798	6132	6714	7248	11,016
ΔC (£)	666	666	666	1056	1484	1000	333	915	1449	5217
Revenues										
$\tilde{h}$, OKS	34.54	**32.46**	**38.70**	34.54	34.54	34.54	34.54	34.54	34.54	34.54
$p*\hat{h}$ (£)	5305	5305	5305	8071	10,912	7958	2653	9309	20,409	62,966
$p*\left(\hat{h}-\tilde{h}\right)$ (£)	301	603	−301	565	904	452	151	529	1159	5216
HRG tariff $\hat{t}$ (£)	6164	5862	6766	6290	6378	6346	5981	6185	6089	5799

*Note:* $h^{*}\left(p=0\right)$ is provider equilibrium post‐surgery health with no bonus; $\underline{h}$ is the level of health over which improving health is costly for the provider; $\tilde{h}$ is the threshold level of health over which the purchaser reimburses the bonus payment; $\hat{h}$ purchaser health objective; $C\left(\hat{h}\right)$ is the cost of treating the patient at the target health objective; ΔC is the difference in provider cost before and after the introduction of the bonus. Scenario I: baseline; Scenarios II and III: lower and higher threshold over which the purchaser reimburses the bonus; Scenarios IV and V: more ambitious objective of the funder; Scenarios VI and VII: lower and higher proportion of fixed costs (relative to baseline); Scenarios VIII and IX: lower and higher level of health over which improving health is costly for the provider. Scenario X: lower proportion of fixe3d costs, higher level of health over which improving health is costly for the provider, and more ambitious target (relative to baseline). The text in bold highlights the change in the key assumptions relative to the baseline model.

Abbreviation: OKS = Oxford Knee Score.

The average cost for a knee replacement across 128 public hospitals (Trusts) in England in 2016–2017 was £5799. The average risk‐adjusted post‐operative health was 35.58 as measured by the Oxford Knee Score. In the baseline calibration (column I of Table A1), we assume that 80% of the costs are fixed, and that the value of post‐operative health above which it is costly to increase health, $\underline{h}$, is equal to 31.5 OKS, just below the minimum post‐operative health observed in the hospital sample (equal to 31.54 OKS). By similar steps as for hip replacement, we re‐cover the cost parameter c=£139.3.

The purchaser chooses a target level of post‐operative health equal to $\hat{h}=36.62$ on the OKS scale, which corresponds to one standard deviation in post‐operative health (equal to 1.04 OKS). Using (23), the price per unit of health improvement (one OKS point) to achieve the objective $\hat{h}=40.55$ is equal to $p^{*}\left(\hat{h}\right)=c\left(\hat{h}-h^{*}\left(p=0\right)\right)=£144.9$. The cost of a knee replacement at the higher post‐operative health is $C\left(\hat{h}\right)=£6465$, and the additional cost relative to pre‐policy intervention is $\Delta C=C\left(\hat{h}\right)-C\left(h^{*}\left(p=0\right)\right)=£666$.

In terms of provider revenues, we assume again that the purchaser sets the level of post‐operative health $\tilde{h}$ (see (1)) over which the purchaser pays for additional health improvements at $\tilde{h}=34.54$, which is equal to the pre‐policy post‐operative health *minus* one standard deviation in post‐operative health. The additional revenues from the bonus are $p^{*}\left(\hat{h}\right)\times \left(\hat{h}-\tilde{h}\right)=£301$. Given that the total cost of a knee replacement is £6565, the basic HRG tariff to ensure that the provider breaks even is $t\left(\hat{h}\right)=£6164$.

The purchaser could instead choose the lower level of post‐operative health threshold $\tilde{h}=32.46$, which corresponds to three standard deviations (rather than one) in post‐operative health (column II in Table A1). Although the price remains unchanged, the provider has now larger revenues from the bonus, equal to £603, and these are accompanied by a lower basic HRG tariff equal to £5862, which ensures again that total revenues are equal to provider costs (£6465).

The purchaser could also set up the scheme as a penalty, by setting the higher threshold of 38.70, which is three standard deviations in post‐operative health above the pre‐policy level (column III in Table A1). The provider now makes a financial loss of −£301. Given that the provider has to break even, the HRG tariff is increased to £6766.

In columns IV and V of Table A1, we assume that the purchaser sets a more ambitious target with a post‐operative health which is respectively 1.5 and 2 standard deviations higher. The price for unit of OKS improvement is now increased, relative to the baseline, to £217.3 and £289.8, respectively.

In columns VI and VII of Table A1 we change, relative to the baseline in column I, the % of costs that are fixed from 80% to 70% and 90%, respectively. The price per unit of health improvement now increases (reduces) to £217.3(£72.4).

In columns VIII and IX, we increase $\underline{h}$ to 32.5 and 33.5, respectively. This implies an increase in the cost function parameter c to £244.4 and £535.9, which translate into a higher price per unit of health improvement equal to £254.2 and £557.3.

Finally, in column X we combine several assumptions that lead to higher prices: the % of fixed costs is 70%, $\underline{h}=33.50$, and the target post‐operative health is two standard deviations above the pre‐policy level. The price per unit of health improvement is £1672.

Overall, the results for knee replacement are qualitatively and quantitatively similar to those obtained for hip replacement.
